# How Silica Surface Chemistry Modulates Interfacial Water: Insights from Machine Learning Molecular Dynamics

**DOI:** 10.1021/acsami.6c00590

**Published:** 2026-05-19

**Authors:** Cong Huy Pham, Margaret L. Berrens, Marcos F. Calegari Andrade, Nir Goldman, Daniel V. Esposito, Tadashi Ogitsu, Tuan Anh Pham

**Affiliations:** † Materials Science Division, 4578 Lawrence Livermore National Laboratory, Livermore, California 94550, United States; ‡ Laboratory for Energy Applications for the Future, 4578 Lawrence Livermore National Laboratory, Livermore, California 94550, United States; § Department of Chemistry and Biochemistry, University of California, Santa Cruz, California 95064, United States; ∥ Department of Chemical Engineering, University of California, Davis, California 95616, United States; ⊥ Chemical Engineering Department & Columbia Electrochemical Energy Center, 5798 Columbia University, New York, New York 10027, United States

**Keywords:** surface chemistry, interfacial water, silica, molecular dynamics simulations, machine learning

## Abstract

Controlling water structure and dynamics at silica interfaces are central to a wide range of technologies, including protective oxide layers for solar water splitting and nanoporous membranes. In this work, we develop a machine learning interatomic potential, trained via active learning, to achieve ab initio accuracy for water confined between hydroxylated silica surfaces over a range of silanol coverages and slit widths. We find that partially hydroxylated surfaces (50 and 75% OH) support stronger water–surface hydrogen bonding and more extended interfacial density profiles than fully hydroxylated (100% OH) surfaces, indicating that increasing OH coverage does not necessarily strengthen interfacial hydrogen-bond networks. Translational diffusion decreases approximately linearly with slit width and OH coverage, whereas rotational dynamics respond nonlinearly. In particular, at the smallest slit width of 5 Å, 75% OH coverage produces an enhanced local tetrahedral ordered interfacial network that strongly suppresses reorientation, while 100% coverage yields a crowded, disordered interfacial layer that also hinders rotation. In contrast, the 50% OH coverage is sufficiently sparse that it does not markedly alter water structure or dynamics under confinement. These results show that coupled control of pore size and surface chemistry enables nonlinear tuning of interfacial water structure and transport, providing a design strategy for optimizing porous silica for either enhanced interfacial stability and controlled reactivity or rapid and selective transport.

## Graphical abstract

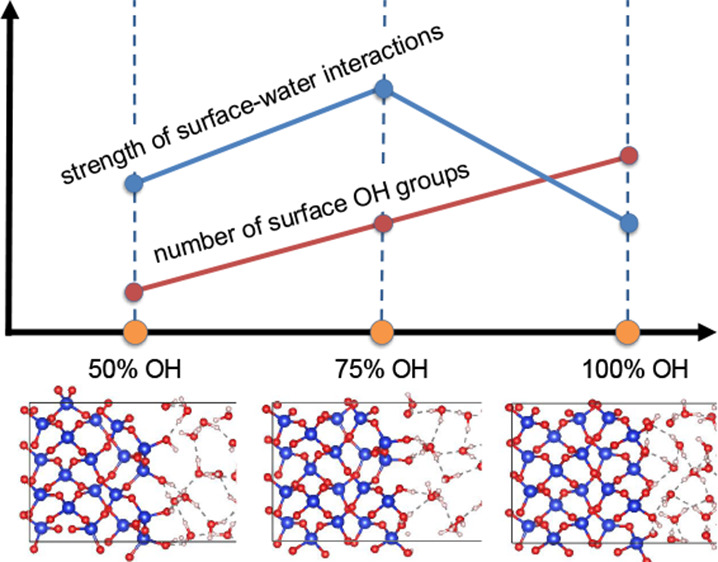

## 1 Introduction

An improved understanding of water properties at the interface with silica is critical for advancing a wide range of environmental,^1–4^ biological,^2,5,6^ and technological fields.^7,8^ As an example, SiO_2_ has been increasingly employed as a protective layer for catalysts for fuel cells and hydrogen production from electrolysis or solar-water-splitting.^9–13^ Silica-based materials have also been explored as thin inorganic proton-conducting membranes in microfabricated fuel cells and related electrochemical devices, where their nanoscale thickness can compensate for higher intrinsic resistivity and make water and proton transport at the SiO_2_ interface a key performance determinant.^14–16^ Beyond improving catalysts durability by mitigating their dissolution and nanoparticle coalescence, the oxide layer–often engineered with nanoporous architectures–can facilitate selective molecular transport between the electrolyte and buried catalysts to enable species selectivity reactivity.^17–21^ In this regard, a deep understanding of how to control transport of water within these porous SiO_2_ layers is paramount for tailoring the oxide to enhance hydrogen production efficiency.

Among the many factors that determine water transport at the interface with SiO_2_, the density and spatial arrangement of surface hydroxyl (−OH) groups are of paramount importance. The −OH groups govern the formation, orientation, and dynamics of the hydrogen-bond network in the adjacent water layer, ultimately affecting wettability, surface charge and interfacial water structure and dynamics. Prior atomistic studies of hydrated amorphous silica—using both empirical force fields and forces derived from density functional theory (DFT)—have shown that surface silanol groups strongly anchor interfacial water via directional hydrogen bonding, producing pronounced orientational ordering of first-layer water relative to the surface, while silanol-depleted regions remain comparatively hydrophobic.^22,23^ Additionally, molecular dynamics (MD) simulations using classical force fields suggest that water molecules in the immediate vicinity of the silica surface–particularly those directly adsorbed via hydrogen bonds to silanol groups–exhibit considerably slower diffusion compared to bulk water.^24^ Classical simulations also suggest a longer residence time for the water molecules found on the first layer on silica surface with high density of hydroxyl groups.^25^

It is also expected that the hydrogen-bond network within porous oxides significantly deviate from that of bulk water, and therefore water structure and dynamics. For example, classical simulations demonstrate that water diffusion is strongly reduced in silica nanopores, with the effect becoming more pronounced in smaller pores where the entire fluid is dominated by nearly immobile interfacial water layers.^24,26^ In larger pores with diameters larger than 2 nm, a core–shell structure emerges whereby a mobile, bulk-like water core coexists with a hindered interfacial shell, resulting in water transport properties that more closely resemble those of the bulk fluid.^26^ Given that H^+^ transport in water depends sensitively on hydrogen-bond network structure and dynamics, confinement-induced deviations from bulk-like water that promote strongly oriented, hydrogen-bond-connected networks are expected to facilitate interfacial H^+^ transport pathways at oxide surfaces.^27–29^ Collectively, existing studies suggest that the interplay between surface chemistry and nanoconfinement has strong effects on transport properties of interfacial water.

Recent advances in machine learning (ML) have enabled the development of new MD simulation paradigms that combine ab initio accuracy with computational efficiency.^30–33^ In this work, we leverage machine learning molecular dynamics (ML-MD) simulations to predict how variations in OH coverage on silica surfaces affect the structure and dynamics of interfacial water. Specifically, we employ the Chebyshev Interaction Model for Efficient Simulation (ChIMES), a physically informed ML interatomic potential (ML-IAP) that combines a multibody cluster-expansion formalism with Chebyshev polynomial representations.^34^ The model was trained via an active learning framework,^35^ iteratively refining training data by sampling configurations from MD simulations and structural optimizations, thereby ensuring robust coverage of configurational space. We also extend our investigation to consider the effects of confinement, which introduces additional complexity by imposing geometric restrictions on water molecules between closely spaced silica layers. It is worth noting that a key motivation for using an MLIP rather than a classical point-charge pairwise force field in this study is its closer fidelity to the underlying electronic-structure reference. Because the MLIP is trained on DFT energies and forces, it can reproduce DFT-consistent interfacial energetics and local forces while retaining the efficiency needed for large-scale sampling. The MLIP also captures many-body, environment-dependent interactions implicitly, which is critical for hydrogen-bonded, highly polar silica–water interfaces where fixed-charge pair potentials can be overly restrictive. In addition, the same MLIP formalism can be extended to chemically heterogeneous surfaces and reactive processes (e.g., proton transfer) without introducing ad hoc bond-breaking rules, which is a focus of our intended future work. Our approach overcomes the limitations of traditional simulation methods, enabling robust predictions of water behavior over a range of hydroxyl coverages and confinement scenarios.

In the following sections, we describe our ML-MD simulation protocol, present our findings on how OH coverage influences the interfacial water structure and dynamics, and elucidate the additional impact of confinement on water properties. In this study, we focus on an ideal α-quartz (0001) surface, which should be viewed as a controlled, well-defined model for assessing the impact of a homogeneous silanol density, rather than as a fully quantitative representation of specific experimental silica materials. Modeling heterogeneous quartz surfaces would require substantially larger surface areas to sample diverse local chemical environments and surface terminations. This, in turn, would necessitate additional active-learning cycles and a significantly expanded DFT training set to maintain MLIP accuracy over a broader configuration space. Such model refinement, and the extension to more structurally and chemically heterogeneous silica surfaces, are part of our intended future work. Our study suggests that increasing OH coverage on SiO_2_ surfaces does not result in a straightforward, linear effects on water structure and dynamics; instead, nonlinear behavior may emerge due to the interplay between enhanced local hydrogen-bond interactions and the cooperative reorganization of the interfacial water network. When water is confined between silica surfaces, these interfacial effects are compounded by geometric restrictions that further modulate water structure and transport properties. Our results provide new insights into the molecular mechanisms governing transport properties of water in porous oxides and open new avenues for the rational design of silica-based materials with tailored surface properties for solar-water-splitting hydrogen production. We note that no dissociated silanols were observed during the simulations, likely because the model does not include water ions such as hydroxide and hydronium. In future work, we plan to extend the MLIPs to include these ions and investigate their impact on surface chemistry.

## 2 Computational Methods

### 2.1 Molecular Dynamics Simulations

The simulation models of water confined in SiO_2_ slit nanopores are illustrated in Figure 1. They comprise SiO_2_ surfaces with three hydroxylation states: 100, 75, and 50% OH coverages, corresponding to 2.0, 1.5, and 1.0 OH groups per surface Si atom in panels (a)–(c), respectively. Starting from the fully hydroxylated surface (100% OH coverage) with eight surface OH groups at each interface, we followed the dehydration mechanisms described in ref 36 to generate quartz surfaces with varying numbers of surface OH groups. The minimum OH coverage considered in this study is 50%, as systems with OH coverages below 50% are not thermodynamically stable based on previous study.^36^

**Figure 1:**
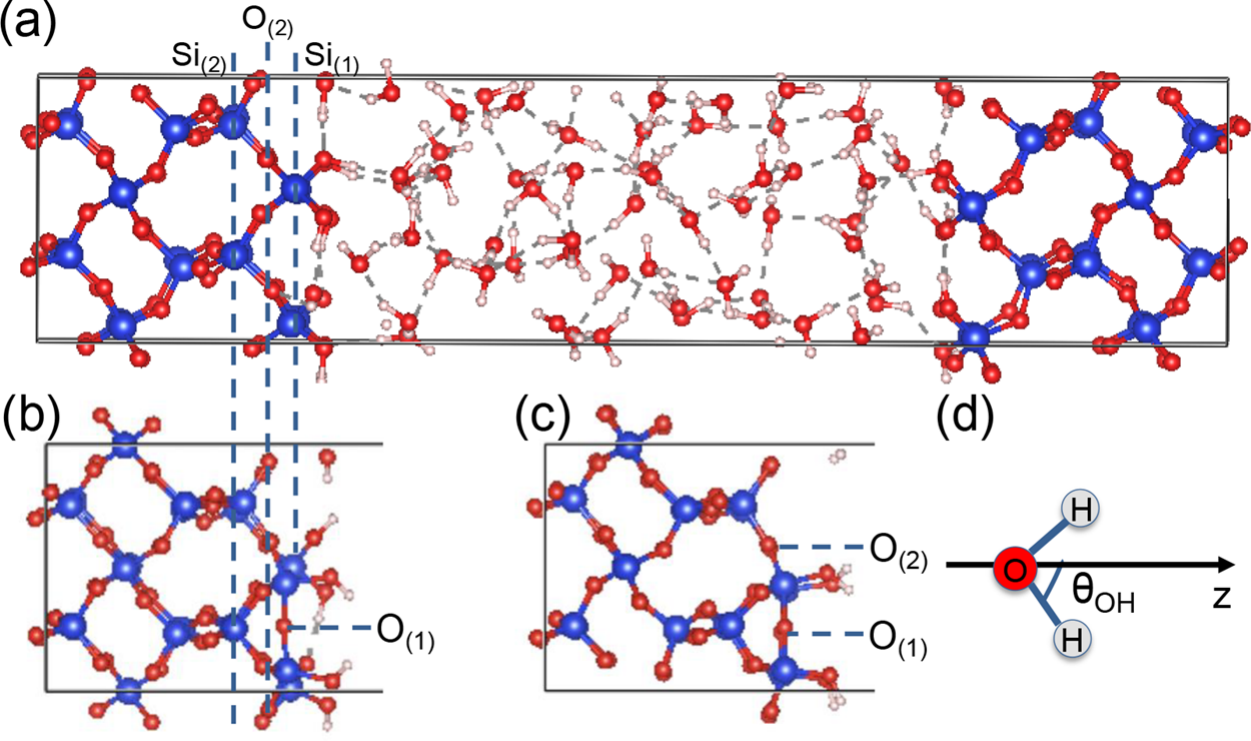
(a) The (0001) α-quartz-water interface system from a snapshot of the simulation. Si atoms are represented in blue, O atoms in red, and H atoms in white. The quartz surface is labeled as “100% OH coverage”, indicating two OH groups bonded to each Si atom at the surface. The “75% OH coverage” and “50% OH coverage” with three and two OH groups for two Si sites at the surface are shown in panel (b) and (c), respectively (d) tilt angle between the O–H bond vector of water and the *z*-axis, which is perpendicular to the quartz surface.

To investigate confinement effects, we consider pore widths of 5, 10, 15, and 20 Å for each surface chemistry; the 5 Å slit represents the extreme-confinement limit, whereas 20 Å approaches bulk-like conditions for the liquid. Following previous theoretical work of confined water between oxide slabs,^29,37^ density in all pores was maintained at 1.0 g/cm^3^, and the lateral cell dimensions were fixed at 9.83 Å × 8.51 Å in the *x* and *y* directions. Our simulation cells contain 174 to 312 atoms, with the largest system of 312 atoms corresponding to 100% surface OH coverage and a pore width of 20 Å.

All molecular dynamics simulations were performed with LAMMPS^38^ in the NVT ensemble at 330 K. The equations of motion were integrated with the velocity-Verlet algorithm using a 0.1 fs time step, and temperature was regulated with a Nose–Hoover thermostat. For each slit width (5, 10, 15, and 20 Å), simulations consisted of 1 ns equilibration followed by a 3 ns production run.

We also performed additional simulations to assess the effects of system size and simulation length. Specifically, we doubled the lateral cell dimensions in both the x and y directions while keeping the surface homogeneous. We find that the structural and dynamical properties are very similar across system sizes and between 2 and 3 ns trajectories (see Figures S1 and S2 in the Supporting Information), indicating that the results reported in this study are well converged with respect to both system size and simulation time.

### 2.2 Machine Learning Potential Development

The ML-IAP used in this work was developed with the Chebyshev Interaction Model for Efficient Simulation (ChIMES), a framework that has been applied to systems involving covalent bonding, reactive chemistry, and phase transformations, including under extreme conditions. In contrast to common neural-network potentials (e.g., Behler–Parrinello networks^39^) and other modern ML-IAPs based on explicit *n*-body expansions such as permutationally invariant polynomials (PiP)^40,41^ or tabulated Gaussian Approximation Potentials, ChIMES represents the total energy as a sum of systematically improvable many-body contributions (typically two-, three-, and four-body terms) with prescribed analytic forms. Specifically, each interaction is expressed using Chebyshev polynomials of transformed interatomic distances over fully connected atomic clusters, which provides fine control over short- and medium-range interactions while retaining physical interpretability. ChIMES potentials have been reported for a variety of condensed-phase systems, including water,^42^ CO,^43^ and HN_3_.^44^ Here, we employ polynomial orders of 12/8/3 for the two-, three-, and four-body terms, respectively, with corresponding cutoffs of 6/5/4 Å. During fitting, atomic force components were weighted by 1.0, while total energies were weighted by 200/*n*_atom_, where *n*_atom_ is the number of atoms in each configuration.

To accurately represent the potential energy surface of the systems, we trained our ML-IAP model using energies and atomic forces obtained from DFT calculations performed with the PWscf code in Quantum ESPRESSO.^45,46^ We used the SCAN functional^47^ due to its robust performance in reproducing the structure and dynamics of liquid water, while also accurately capturing interactions between water and metal oxide surfaces.^48–50^ A kinetic energy cutoff of 110 Ryd and a charge density cutoff of 440 Ryd were employed. The DFT-MD equations of motion were numerically integrated using the Verlet algorithm^51^ with a time step of 0.5 fs. The canonical ensemble was sampled using a single chain of three Nose Hoover thermostats^52,53^ coupled to the ionic degrees of freedom. The temperature was set to 330 K, and the simulation time was approximately 5 ps. Initial training data were generated by uniformly sampling configurations from DFT MD trajectories, after which active learning was used to select the most relevant atomic configurations. The final model was trained on 961 structures. The validation root-mean-square errors for energies and forces were 0.0026 eV per atom and 0.16 eV/Å, respectively.

The choice to study this system with DFT rather than a fixed point-charge model is due to the need to account for interfacial polarization interactions of the surface with water. To highlight the importance of explicitly capturing interfacial polarization, we analyzed 15 representative frames from the 5 and 20 Å systems and computed water dipole moments using Quantum ESPRESSO (with the same parameters described above) and Wannier centroids as described in Calegari et al.^54^ (as shown in Figure S3 in the Supporting Information). In the 20 Å system, the dipole distribution in the bulk-like region is centered near 2.9 D, as expected. However, at the interface, and throughout the 5 Å system, the distribution shifts to higher values. This clear enhancement of dipole moments at confinement interfaces demonstrates significant interfacial polarization, underscoring the need to explicitly account for these effects, as they directly influence structural and dynamical properties.

To further validate the developed ML-IAP, we compare its predicted structural properties against those obtained from DFT-MD simulations. For illustration, Figure 2 shows the results for systems with 100% OH coverage; corresponding figures for systems with 75 and 50% OH coverage are provided in the Supporting Information (Figures S4 and S5). In our comparison, we examine the radial distribution functions (RDFs) for various atomic pairs, including water–water interactions (Ow–Ow, Hw–Hw) and water–surface interactions (Ow–Os, Hw–Os, etc.). A good agreement between the DFT and ChIMES RDFs across most pairs indicates that the ML-based force field accurately captures the essential structure of interfacial water, reproducing both the short-range ordering within the first few angstroms and the broader oscillatory decay characteristic of density layering. Only minor deviations in certain RDF peaks were observed, for instance, in the Ow–Hs RDF. Overall, Figure 2 highlights the reliability of the ChIMES potential in capturing the local structure at the silica–water interface and underlines how variations in OH-termination (i.e., surface chemistry) can tune interfacial hydration environments.

**Figure 2:**
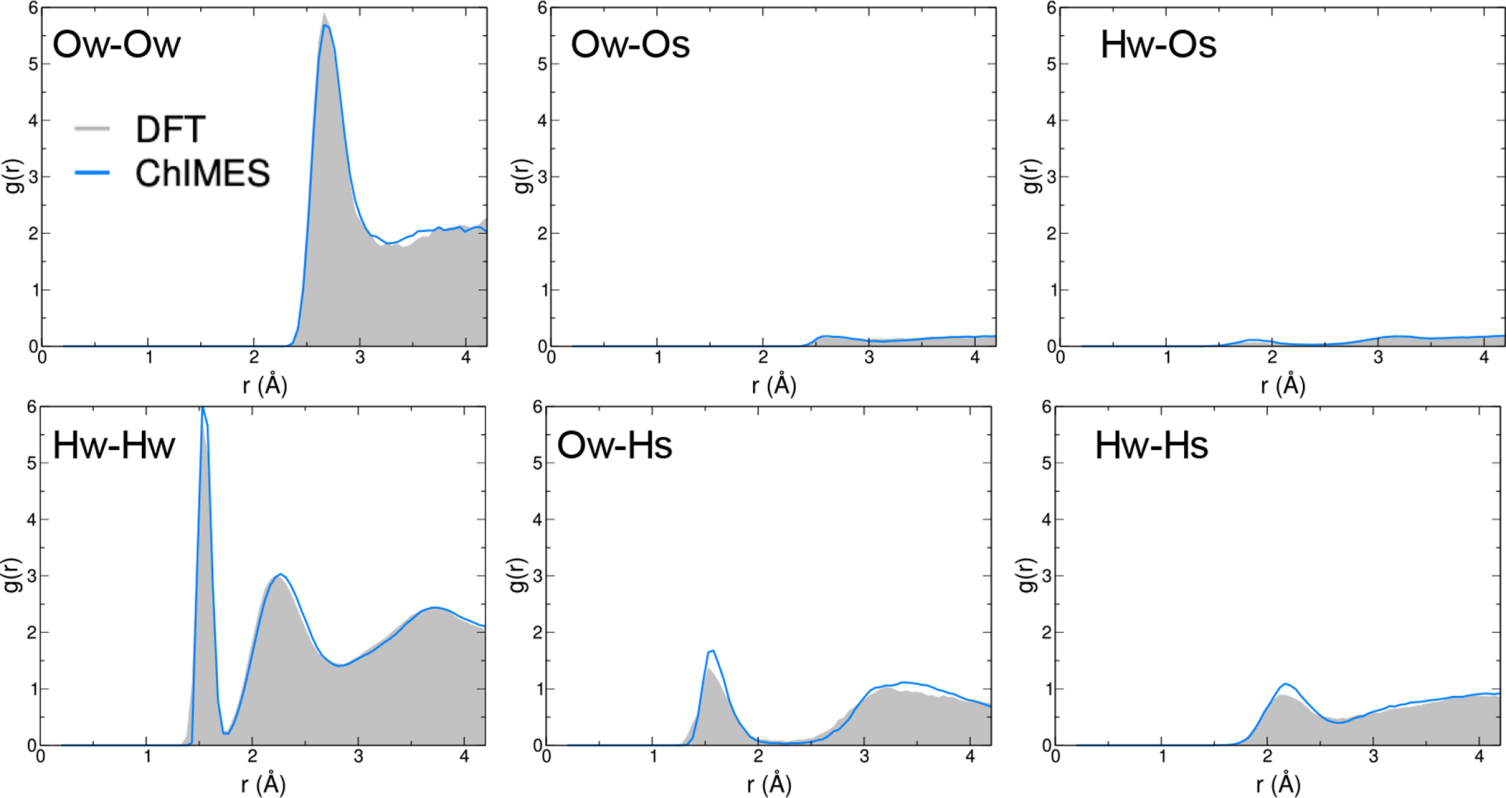
Predicted radial distribution functions g­(r) by DFT and ChIMES for different O–O, H–H and H–O pairs. Subscripts “w” and “s” indicate the atoms belong to “water” and “surface”, respectively.

## 3 Results

### 3.1 Effects of the Surface Chemistry

We start by discussing the effects of surface chemistry in shaping the structure and dynamics of water. On a fully hydroxylated quartz surface, each Si atom is bonded to two OH groups with oxygen atoms labeled as O_(2)_ in Figure 1, and the confined water molecules primarily interact with these OH groups. To investigate the effect of surface chemistry on confined water, we first examine how varying surface silanol densities influence the system at the largest slit width studied. Shown in in Figure 1, in the system with 75% OH coverage, two types of surface Si atoms are present: one type is bonded to two OH groups (labeled as Si_(2)_), similar to the fully hydroxylated case, while the other type is connected to a single OH group and is bridged to another Si atom via an O_(1)_ atom (labeled as Si_(1)_). On the other hand, for the surface with a 50% OH coverage, each surface Si atom is bonded to one OH group and is also linked to neighboring Si atoms through the bridge O_(1)_ atom. Accordingly, in both the 75% and 50% OH coverage systems, confined water molecules can interact not only with the OH groups but also strongly with the bridging O_(1)_ and O_(2)_ atoms, resulting in a more complex interfacial environment.

Figure 3a–c presents the atomic number density profiles of oxygen atoms in water molecules along the *z*-direction for systems with varying OH coverages. The number of water molecules is kept constant across all surface systems considered. For the system with 100% OH coverage, water molecules remain restricted above the surface oxygen sites in the *z*-direction (see Figure 1a) due to the strong intrasurface hydrogen bond network, resulting in a pronounced peak in the oxygen density near the surface. In contrast, the intrasurface hydrogen bond networks in systems with 75 and 50% OH coverage are weaker because of the reduced number of OH groups. As a result, water molecules are able to penetrate further into the surface region and form hydrogen bonds with the O_(1)_ and O_(2)_ atoms.

**Figure 3:**
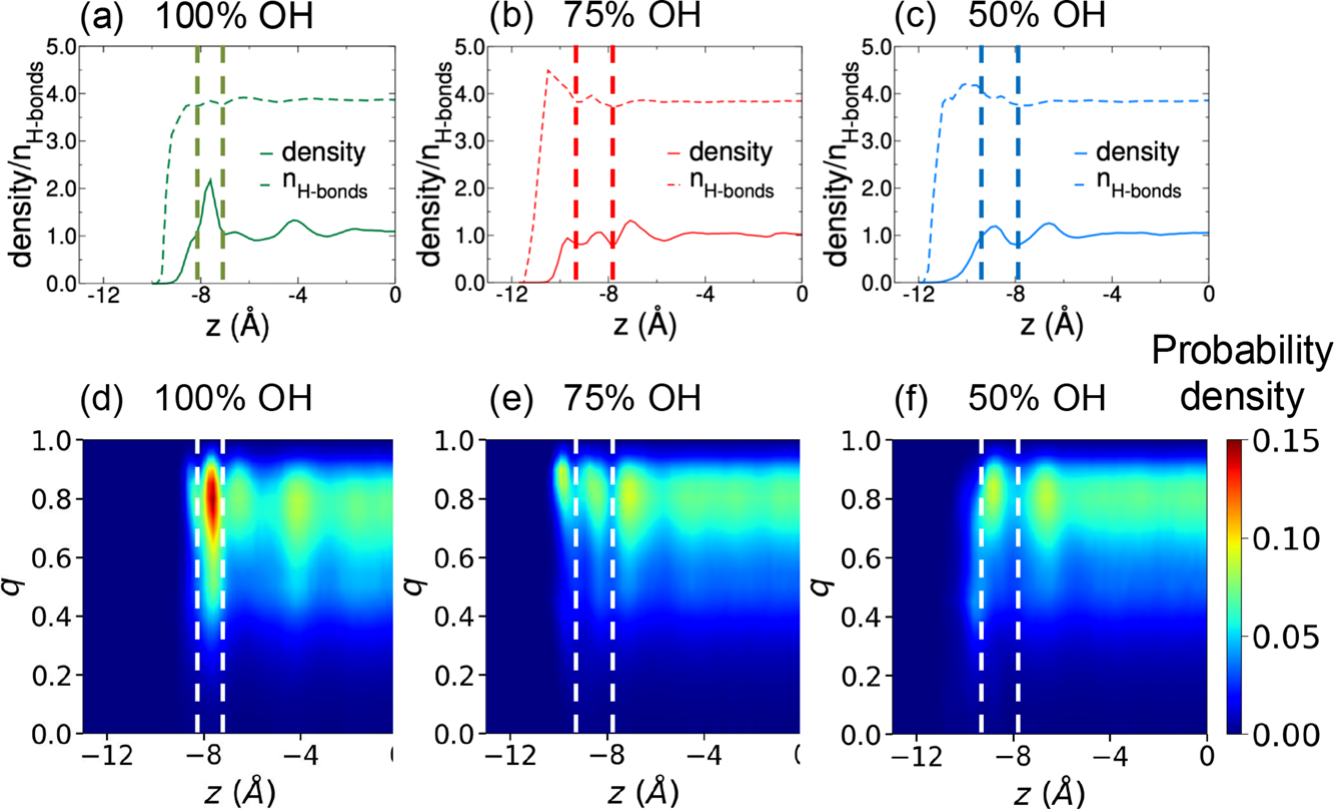
(a–c) Atomic number density profiles of oxygen atoms in water molecules along the *z*-direction, shown relative to the experimental bulk liquid water density at 300 K and 1 bar, for various SiO_2_ surface systems with a water layer thickness of *L* = 20 Å. The number of hydrogen bonds per water molecule, including both water–water and water–surface hydrogen bonds, is also shown (dashed lines). (d–f) Distributions of the tetrahedral order parameter along the *z*-direction for the corresponding systems. The color bar indicates the probability density of the tetrahedral order parameter *q*. Oxygen atoms of silanols are considered in the tetrahedral order parameter calculation for waters at the interface. The dashed lines separate different water layers relative to the surface, and they are the same for the systems with 75 and 50% OH coverage.

This difference in water penetration and proximity to the surface directly influences the hydrogen bonding environment of the confined water. Hydrogen bonds between water molecules were identified using a standard geometric criterion, where two molecules are considered hydrogen bonded if the O–O distance is less than 3.5 Å and the O–H···O angle is greater than 150°.^55^ For the 100% OH coverage system, the total number of hydrogen bonds near the surface (that water molecules participated in) is slightly lower than that in bulk water, as the surface OH groups form a strong hydrogen bond network among themselves. In contrast, for the systems with 75 and 50% OH coverage, an increased number of hydrogen bonds for interfacial water is seen compared to bulk water. This enhancement is most pronounced at 75% OH coverage, followed by 50%, and is weakest for the fully hydroxylated surface. Surprisingly, these results indicate that the solvation shell of interfacial water is strongest at 75% OH coverage and weakest at 100% OH coverage. This trend is further supported by the analysis of structural properties, such as the RDF of the Ow–Os pair shown in the Supporting Information (figure S6), where the ordering of the strength of surface water interactions follows 75% > 50% > 100% OH coverage, as attributed to the intensity of the first peak in the RDF.

To provide direct insights into the underlying structure of confined water, we also investigate the evolution of structural order parameters. Our focus is on the tetrahedral order parameter *q*, defined as

$$q=1-\frac{3}{8}\overset{3}{\underset{j=1}{\sum }}\overset{4}{\underset{k=j+1}{\sum }}{\left(\cos\psi_{jk}+\frac{1}{3}\right)}^{2}$$ 1

where ψ is the angle between the vectors connecting the oxygen atoms of the central water molecule and two of its four nearest neighbors, *j* and *k*. When *q* = 1, the water molecules are in perfect tetrahedral (ice-like) coordination, whereas *q* = 0 indicates that the water molecules are randomly oriented, similar to an ideal gas. In our calculation of the tetrahedral order parameter *q*, we treated surface silanol oxygen atoms as potential hydrogen-bonding partners. Notably, water–silanol interactions are similar in character to water–water interactions in confined water, as evidenced by the nearly identical first peak in the oxygen–oxygen radial distribution functions for water–surface and water–water pairs (Figure S6 in the Supporting Information). Therefore, the same definition of the tetrahedral order parameter used in previous studies remains appropriate for quantifying local water structure in our system.

The distributions of *q* along the *z*-direction for different surfaces are presented in Figure 3d–f. For all three surface densities, the bulk-like region of the confined water exhibits a *q* distribution centered around *q* = 0.75. As shown in Figure 3d for the fully hydroxylated SiO_2_ nanopore, the *q* distribution indicates that the first interfacial water layer is highly structured with a peak centered at *q* = 0.8. In the second water layer, the distribution becomes broader, featuring a high-water density peak near *q* = 0.8 accompanied by a long tail extending toward lower values around *q* = 0.45. This behavior reflects the coexistence of predominantly structured water with a smaller population of more disordered water molecules.^56,57^ These findings are consistent with the previous studies,^58^ which reported ice-like and liquid-like characteristics in the first and second interfacial water layers, respectively.

Examining the effect of surface chemistry on water ordering, the 75% OH coverage surface exhibits the highest degree of interfacial ordering observed in this study. In this case, the first water layer displays a sharp peak in the *q* distribution at *q* = 0.85, indicating a strongly ordered structure. The second water layer also exhibits a peak at a *q* value exceeding that of bulk-like water (*q* = 0.81), confirming enhanced ordering across the first solvation layers. Unlike the 100% case, this second layer does not exhibit a significant low-*q* tail below *q* = 0.7. In contrast, for the 50% OH coverage surface, the first and second water layers merge into a single interfacial layer. This layer exhibits a *q* distribution with a peak at *q* = 0.81, comparable to the second solvation layers observed in the 75% case, and similarly lacks a pronounced low-*q* tail. Taken together, these results demonstrate that surface chemistry strongly modulates the extent and spatial persistence of interfacial water ordering, leading to distinct solvation-layer structures that deviate markedly from bulk-like behavior.

Next, we investigate the orientation of interfacial waters for SiO_2_ surfaces with different OH coverages. Figure 4 shows the distributions of cos θ_OH_ for the first two interfacial water layers for systems with different surface OH coverages, where θ_OH_ is the tilt angle of water OH bonds relative to the *z*-axis. In the first layer of water near the fully hydroxylated surface (Figure 4a), there are two peaks in the distribution centered at cos θ_OH_ = 0.625 and cos θ_OH_ = −0.6, corresponding to two types of orientation with θ_OH_ = 51° and θ_OH_ = 127°. These values differ from a previous study,^58^ where the angles of θ_OH_ = 15° and θ_OH_ = 105° were reported. The discrepancies can be attributed to the use of different computational models; DFT-MD simulations in the previous study^58^ utilized the GGA Becke-Lee–Yang–Parr functional,^59,60^ while MD simulations in this study employed ML-IAP fitted to the metaGGA SCAN functional.^47^ It is also important to note that the time scale of the MD simulations in this study extends to several nanoseconds, which is significantly longer than the DFT-MD simulations in ref 58, where only a few picoseconds of simulation were performed. Nanosecond time scales are typically required to achieve statistical convergence of transport properties, such as the diffusion coefficient of water.^61^ In the second water layer, some OH groups are oriented toward the water molecules in the first layer, corresponding to cos θ_OH_ = −1.0, or θ_OH_ = 180°. The orientation of the other OH in the second water layer is more flexible, with the peak in the distribution centered at cos θ_OH_ = 0.25, which corresponds to θ_OH_ = 75°. These findings are in good agreement with a previous study,^58^ where the reported values were θ_OH_ = 165° and θ_OH_ = 75°.

**Figure 4:**
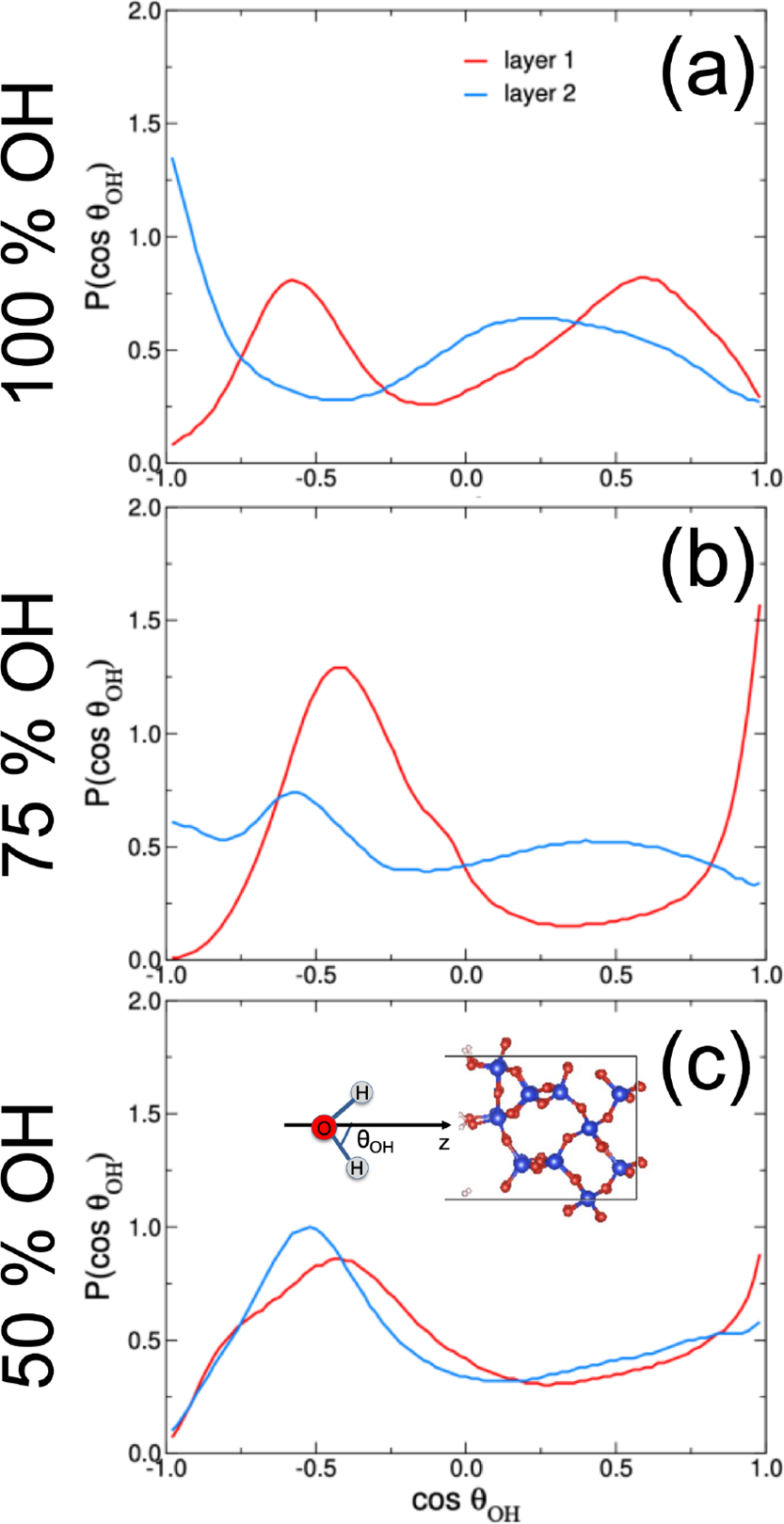
Distribution of cosθ_OH_ for the first two interfacial water layers, where θ_OH_ denotes the tilt angle between the O–H bond vector of water and the *z*-axis, which is perpendicular to the quartz surface (see Figure 1d). Systems with 100% OH, 75% OH, and 50% OH coverages are considered, each with a water thickness of *L* = 20 Å.

As with the water ordering the surface chemistry significantly alters the orientations of interfacial waters. Shown in Figure 4b,c for both 75 and 50% surfaces, there is a peak in the distribution at *cos* θ_OH_ = 1.0, corresponding to OH groups pointing away from the surface. This peak is absent in the 100% OH coverage system. Additionally, there is a peak distribution centered at *cos* θ_OH_ = −0.4. Compared to the 100% system, in the 75% system, the second water layer shows an additional peak distribution at cos θ_OH_ = −0.55 due to the more complex surface environment. This peak is still present in the second water layer of the 50% system, but the peak at cos θ_OH_ = −1.0 is no longer present. In the 50% system, the distributions for the first and second water layers are very similar, indicating that a single layer would be sufficient to describe the interfacial water structure.

At the molecular level, these orientational trends reflect how the balance between water–surface and water–water hydrogen bonding evolves with surface hydroxyl coverage. For the fully hydroxylated surface, the chemically uniform and densely packed OH groups impose preferred orientations on interfacial water in the first layer, as evidenced by two dominant orientational populations. However, this orientational constraint does not persist into the second solvation layer, where a combination of strongly downward-oriented OH groups and a broad distribution spanning intermediate angles indicates increased orientational flexibility and partial disruption of layer-specific ordering. As the OH coverage is reduced, the surface becomes increasingly heterogeneous, altering the available hydrogen-bonding motifs at the interface. At intermediate coverage (75%), this leads to a stronger and more coherent orientational bias in the first layer, accompanied by a broader but correlated distribution that extends across orientations pointing away from the surface, consistent with a more uniformly connected interfacial hydrogen-bond network. In the 50% OH coverage case, the similarity of the orientational distributions in the first and second water layers indicates a diminished layer-by-layer structural differentiation, consistent with a more bulk-like hydrogen-bond network extending closer to the surface.

Differences in surface chemistry, which lead to variations in the structural properties of interfacial water, are also reflected in the vibrational spectra. The calculated vibrational density of states (vDOS) for hexagonal ice (*I*_h_), liquid water, and surface water with varying OH coverages are presented in Figure 5. Unlike the findings in ref 58, our vDOS calculations do not show significant ice-like features in the surface water (top panel). Across all systems with different OH coverages, the vDOS in the vibrational stretching region exhibits a bimodal distribution. The two frequency modes correspond to OH stretching: one at 3500 cm^–1^, characteristic of water–water interactions as in liquid water, and another at 3700 cm^–1^, associated with water-surface interactions as seen in previous experimental vibrational sum-frequency spectra of a water-quartz interface.^62^ The presence of the SiO_2_ surface disrupts the hydrogen-bond network of liquid water, resulting in the emergence of this higher-frequency vibrational mode. As expected, the features in the vDOS directly correlate with the structural order of interfacial water illustrated by the Ow–Os RDF shown in Figure 5, lower panel. The peak at 3700 cm^–1^ is less pronounced in the 75% system, as it shows the highest structural order of water near the interface. The 100% system exhibits the least ordered interfacial water, and we observe the most pronounced peak at 3700 cm^–1^. Du et al.^62^ suggested that for a solid surface, the restriction of packing surface water molecules against a rigid wall forces the molecular arrangement into a more ordered structure. Such that, ordered water structure corresponds to a more hydrophilic surface. One would expect then that a higher silanol density would give a more ordered water structure but in this work we see instead a nonlinear trend of ordering and hydrophobicity for the silanol densities sampled. The O–H stretching mode as a function of silanol surface density can have a direct correlation with macroscopic descriptors such as wettability or contact angle. Specifically, the red shift of the interfacial O–H stretching mode for the 75% case, reflects stronger hydrogen bonding between water and the surface, which lowers the solid–liquid interfacial free energy and, via Young’s equation, could correspond to a reduced contact angle and enhanced wettability as was seen in previous studies at a water graphene interface.^63^

**Figure 5:**
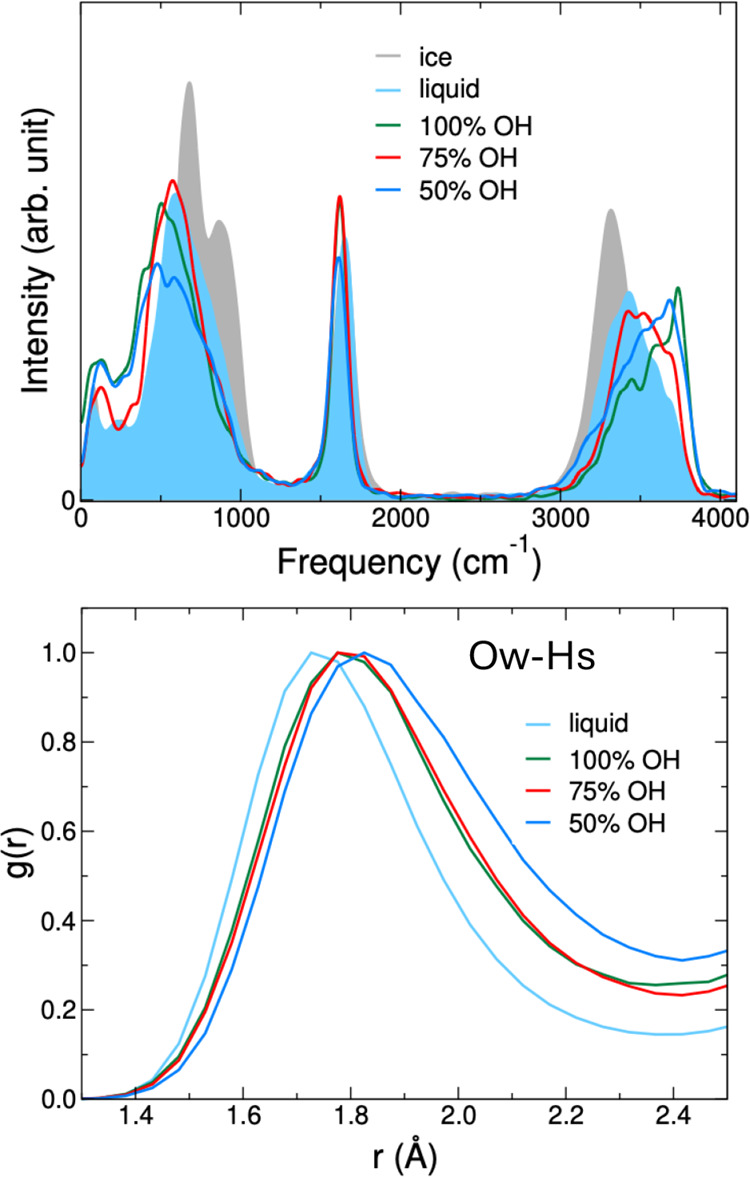
Top: Calculated vibrational density of states (vDOS) for hexagonal ice (*I*_h_), liquid water, and surface water in systems with varying OH coverages. Only OH bonds from water molecules are included in the analysis. Bottom: Calculated Ow-Hs RDF for surfaces with different OH coverages, along with the Ow–Ow RDF for liquid water.

Taken together, the orientational, structural, and vibrational analyses demonstrate nonlinear effects of surface chemistry in controlling the organization of interfacial water. Variations in OH coverage modulate the balance between water–surface and water–water hydrogen bonding, which in turn governs both the orientational constraints and the degree of tetrahedral ordering near the interface. At intermediate OH coverage (75%), interfacial water exhibits the strongest orientational constraints and highest tetrahedral order, consistent with a more uniformly connected hydrogen-bond network extending across the first two solvation layers. This enhanced structural order is accompanied by a reduced contribution from the high-frequency (∼3700 cm^–1^) OH stretching mode in the vDOS, indicating stronger interfacial hydrogen bonding. In contrast, the fully hydroxylated surface (100%) promotes greater orientational heterogeneity and reduced tetrahedral order, reflected in a more pronounced 3700 cm^–1^ vibrational feature associated with the weakest hydrogen-bonding at the interface. At low OH coverage (50%), diminished surface templating leads to broader orientational distributions, weaker layer differentiation, and vibrational signatures in the OH-stretching region in between that of the 100% and 50% cases. Together, these results demonstrate that interfacial water structure and dynamics are maximally ordered at intermediate OH coverage, while both higher and lower coverages favor increased orientational freedom and disrupted hydrogen-bond networks.

### 3.2 Effects of Nanoconfinement

Building on prior studies showing that interfacial water transport in SiO_2_ systems is strongly influenced by both the density and spatial arrangement of surface hydroxyls, with water diffusion markedly reduced in silica nanopores,^58^ we now examine how the pronounced, nonlinear dependence of structural properties on surface chemistry evolves with decreasing slit width, and how this evolution influences diffusivity. Figure 6 presents the surface water diffusivity as a joint function of silanol density and slit width. As expected, across all surface densities, diffusivity decreases nearly monotonically with stronger confinement, such that at the smallest slit width of 5 Å water diffusion becomes minimal or effectively suppressed. Diffusivity also decreases monotonically (except for the 20 Å case) with increasing surface density which is consistent with earlier MD and experimental results.^64^ It is worth noting that the experimental measurements in ref 64 were performed using Overhauser Dynamic Nuclear Polarization (ODNP) spectroscopy to enhance signal intensity in a low-field NMR experiment. In those experiments, the water was not confined, and the silica surface was not as homogeneous as the idealized surfaces considered in our study. Our joint analysis indicates that confinement acts synergistically with surface chemistry, such that reduced slit width significantly enhances the retardation of surface water dynamics.

**Figure 6:**
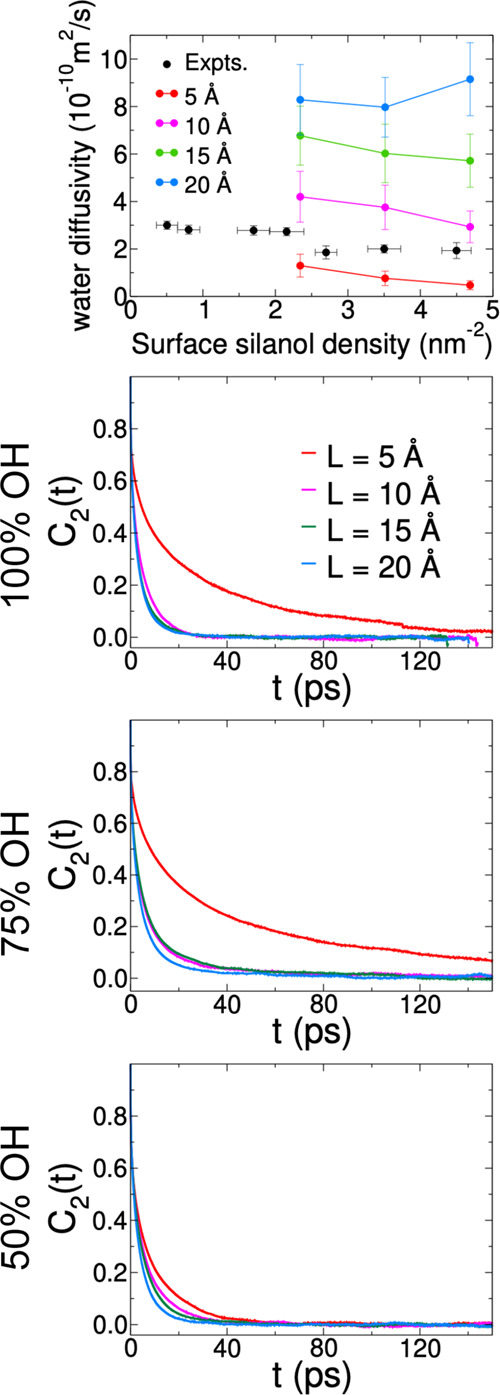
Top panel: Confined water diffusivity for the same systems, compared with experimental data in ref 64. Bottom three panels: Orientational correlation function for the rotation of confined water molecules in systems with 100, 75, and 50% OH coverages, and confined water thicknesses of L = 5, 10, 15, and 20 Å.

Figure 6 also shows the orientational correlation functions describing rotational dynamics of the confined water. At the lowest silanol coverage (50%), confinement has only a minor influence on rotational relaxation. In contrast, for 75 and 100% coverages, rotational dynamics remain largely unchanged at slit widths of 20, 15, and 10 Å but exhibit a pronounced slowdown at 5 Å indicating a significant confinement-induced restriction on water reorientation. Despite the roughly linear decrease in translational diffusivity from 50 to 100% surface coverage, the rotational dynamics behave quite differently. Notably, the fact that only the 75 and 100% coverages exhibit such a marked reduction in reorientation under extreme confinement is unexpected, suggesting a nontrivial interplay between surface chemistry and confinement effects on water rotational dynamics.

To better understand the significant confinement-induced restriction on water reorientation, we examine the structure of the SiO_2_ surface at the smallest slit width sampled. Figure 7 shows snapshots of the systems for the three surface densities, along with the Hs–Hs and Hs–Os RDFs for the 5 Å case. The Hs–Hs RDF indicates that at 50% surface density there are essentially no Hs–Hs interactions, as reflected by the absence of a peak at 2.5 Å. As the surface silanol density increases, a peak begins to appear at 2.5 Å, with the highest intensity observed at 100% coverage. A similar trend is seen in the Hs–Os RDF where at 1.75 Å no peak is present for the 50% case, but as the silanol density increases a peak emerges and becomes strongest for the 100% surface. These RDFs help rationalize the significant confinement-induced restriction on water reorientation for the 75 and 100% cases. They show that beyond a surface density of 50% the emergence of surface structural interactions at 1.75–2.5 Å marks the onset of interfacial crowding, which corresponds to the observed slowdown in water reorientation and diffusion. In contrast, at 50% surface density the surface OH groups remain sufficiently spaced to allow water molecules to rotate freely. This overcrowding effect is also evident in the snapshots shown in Figure 7. At low surface density, no surface–surface interactions are present, but as the density increases, these interactions become increasingly pronounced, marking the onset of overcrowding. This effect is most apparent for the 5 Å case, where the slit is narrow enough that water diffusion and rotation are governed almost entirely by surface contributions. As the slit width increases, however, a growing fraction of the water behaves more “bulk-like,” diminishing the relative influence of surface chemistry and surface density.

**Figure 7:**
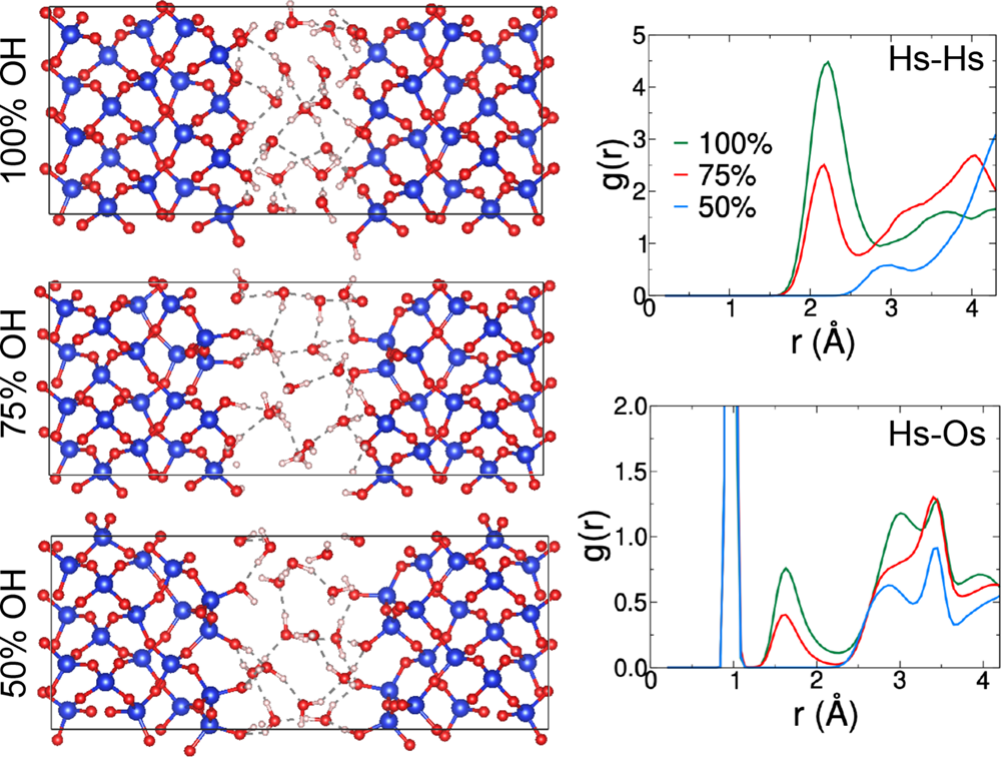
Left: MD snapshots of systems with 5 Å pore sizes and varying OH coverages considered in this study. Right: Corresponding Hs–Hs and Hs-Os RDFs.

To further examine how confinement influences transport, we next consider its effect on the structure of the confined water itself. Figure 8 reports the probability distributions of the tetrahedral order parameter for water as a function of surface density and slit width. For the 50% surface density case, the distribution is nearly insensitive to confinement, consistent with the trends observed for water transport. At 75% coverage, the distribution also remains unchanged except at 5 Å, where it shifts toward higher *q* values, indicating a more ordered water structure. In contrast, at 100% surface density the distribution systematically shifts to lower *q* values with increasing confinement, reflecting a progressively more disordered water network. This leads to an unexpected result at 5 Å – water becomes more ordered at 75% coverage but more disordered at 100% coverage relative to the 50% case. Comparing with the 5 Å snapshots in Figure 7, we find that 75% coverage appears to create a surface environment that allows interfacial water to form favorable hydrogen-bond configurations (with enhanced local tetrahedral ordered) when the system is dominated by surface interactions. However, increasing the coverage to 100% induces overcrowding, characterized by a pronounced rise in surface–surface interactions, which prevents the formation of these optimal hydrogen bonds between water and the surface. The result is a significantly more disordered water structure. Together, these structural trends help explain the confinement-induced changes in rotational transport: the highly ordered network at 75% restricts rotational mobility by locking water molecules into stable hydrogen-bond geometries, whereas the overcrowded and disordered environment at 100% slows rotation through steric hindrance and frequent frustrated reorientations.

**Figure 8:**
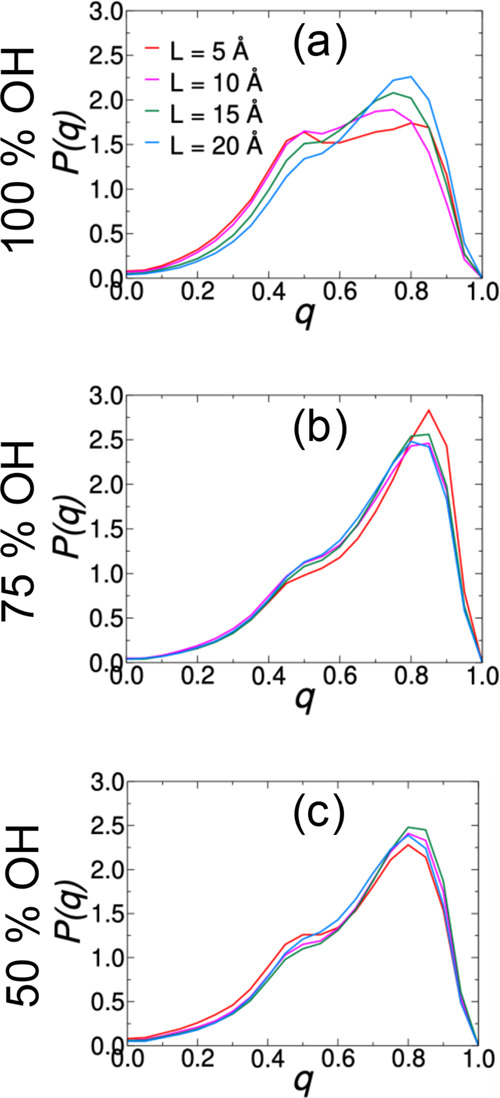
Distribution of the tetrahedral order parameter for systems with 100, 75, and 50% OH coverages, and water thicknesses of L = 5 Å, 10 Å, 15 Å, and 20 Å. Oxygen atoms of silanols are considered in the tetrahedral order parameter calculation for waters at the interface.

## 4 Conclusions

In conclusion, we identify and discuss nonlinear effects in the structure and transport of water confined within SiO_2_ nanopores as a function of silanol surface density and slit width. Considering first the influence of surface chemistry at the largest sampled slit width, we find that the 50 and 75% OH coverage systems exhibit stronger water–surface interactions than bulk water, while the fully hydroxylated (100%) surface supports a comparatively weaker interfacial hydrogen-bond network. Consistent with this behavior, the water density profiles for the 50 and 75% OH coverages extend further into the interfacial region than those observed for the 100% OH system. These results reveal a counterintuitive trend: increasing the density of surface hydroxyl groups does not necessarily enhance interfacial hydrogen bonding with confined water. Given the strong sensitivity of proton transport to hydrogen-bond connectivity and orientational order, the well-connected hydrogen-bond network formed at 75% OH coverage under strong confinement is expected to facilitate efficient proton hopping, whereas the overcrowded and disordered interfacial environment at 100% OH coverage may impede coherent proton transfer despite strong surface interactions; however, quantifying these effects will require further experimental and theoretical studies. These findings are broadly consistent with prior work on water-splitting materials and interfacial systems, including ab initio studies of doped electrocatalysts and recent machine learning-driven simulations of hydrated interfaces, which similarly emphasize the importance of local structure and solvent environment^32,65–67^

The nonlinear trends in interfacial water structure and dynamics demonstrate that confinement and surface chemistry can be used to tune the local solvent environment. While we do not explicitly model proton transport or reaction kinetics, the observed changes in water relaxation and hydrogen-bond connectivity serve as indirect descriptors of solvent reorganization preceding proton-coupled processes. In this context, faster dynamics (e.g., for pores >5Å with lower OH coverage) suggest more labile hydrogen-bond networks that may facilitate proton transport, whereas smaller pores (<1 nm) restrict ion permeation. These implications are qualitative but establish a framework for linking nanoscale water dynamics to transport and selectivity in confined systems.

## Supplementary Material



## Notes

Oxygen–oxygen radial distribution functions, confined water diffusivity, kernel density estimate, and predicted radial distribution functions (PDF)
