# Supplementary material for: How Silica Surface Chemistry Modulates Interfacial Water: Insights from Machine Learning Molecular Dynamics
Source: ACS Appl Mater Interfaces. 2026 May 19;18(21):30456–66. doi: 10.1021/acsami.6c00590 (PMC13244366; doi:10.1021/acsami.6c00590)
Supplement: Supplementary file 1 [file am6c00590_si_001.pdf]

Supporting Information: How Silica Surface Chemistry Modulates Interfacial Water: Insights from Machine Learning Molecular Dynamics

Cong Huy Pham,[*,†] Margaret L. Berrens,[†,‡] Marcos F. Calegari Andrade,[¶] Nir Goldman,[†,§] Daniel V. Esposito,[‖] Tadashi Ogitsu,[*,†,‡] and Tuan Anh Pham[*,†,‡]

†*Materials Science Division, Lawrence Livermore National Laboratory, Livermore, California 94550, United States*

‡*Laboratory for Energy Applications for the Future, Lawrence Livermore National Laboratory, Livermore, California 94550, United States*

¶*Department of Chemistry and Biochemistry, University of California, Santa Cruz, California 94550, United States*

§*Department of Chemical Engineering, University of California, Davis, California 95616, United States*

‖*Chemical Engineering Department & Columbia Electrochemical Energy Center, Columbia University, New York, New York 10027, United States*

E-mail: pham20@llnl.gov; ogitsu1@llnl.gov; pham16@llnl.gov

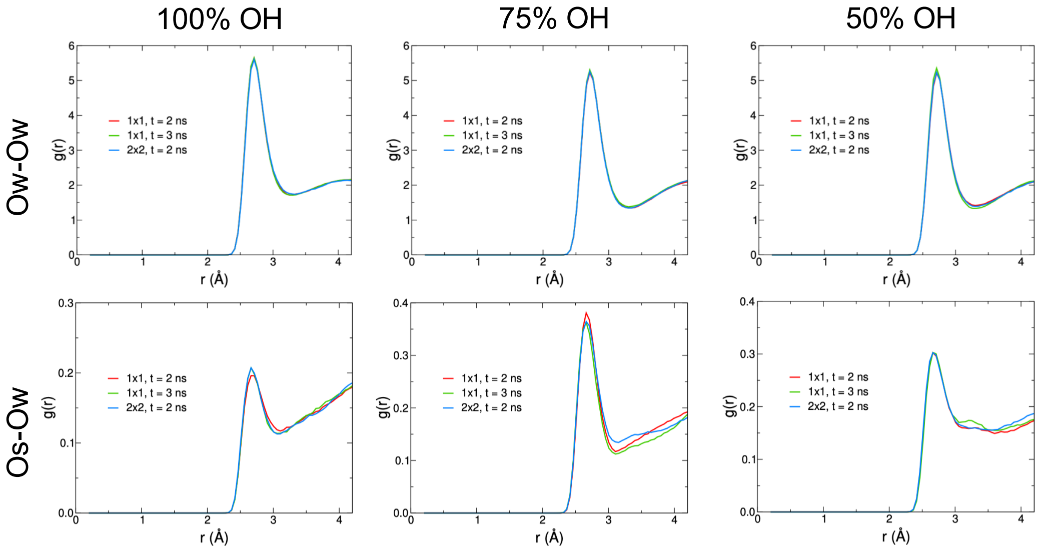

Figure S1: Oxygen-oxygen radial distribution functions (RDFs) for water-water and water-surface interactions, comparing simulations across different system sizes and simulation durations.

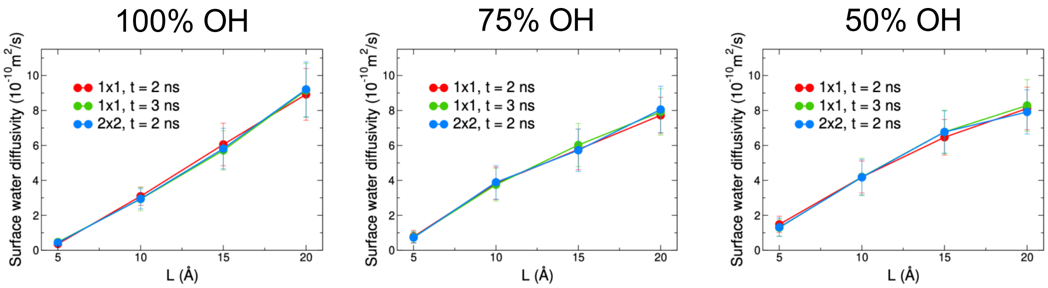

Figure S2: Confined water diffusivity for systems with 100%, 75%, and 50% OH coverages and water thicknesses of L = 5 Å, 10 Å, 15 Å, and 20 Å, comparing simulations across different system sizes and simulation durations.

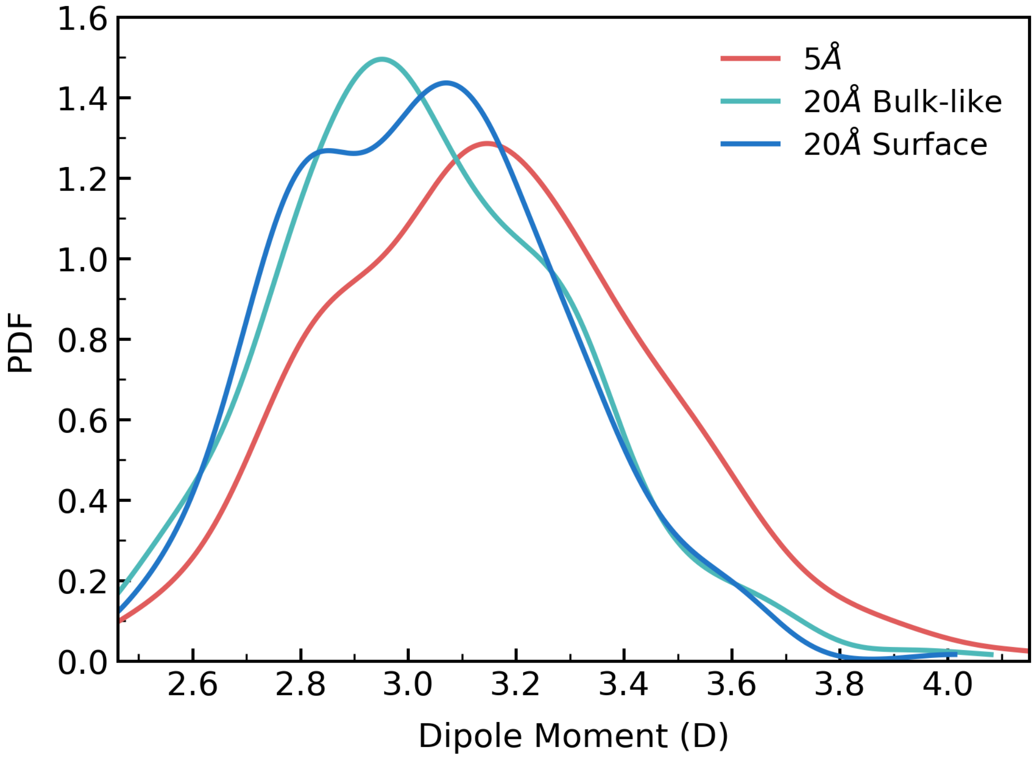

Figure S3: Kernel density estimate of the probability distribution of the dipole moments of the water molecules in the system sampled from 15 frames from the 5 and 20 angstrom confinement systems. For the 20 Å case the surface water molecules were chosen within a cutoff determined in Figure 3. Frames from both cases are sampled from the 100 % silanol surface density cases. Bulk liquid water has a total dipole moment of 2.9.

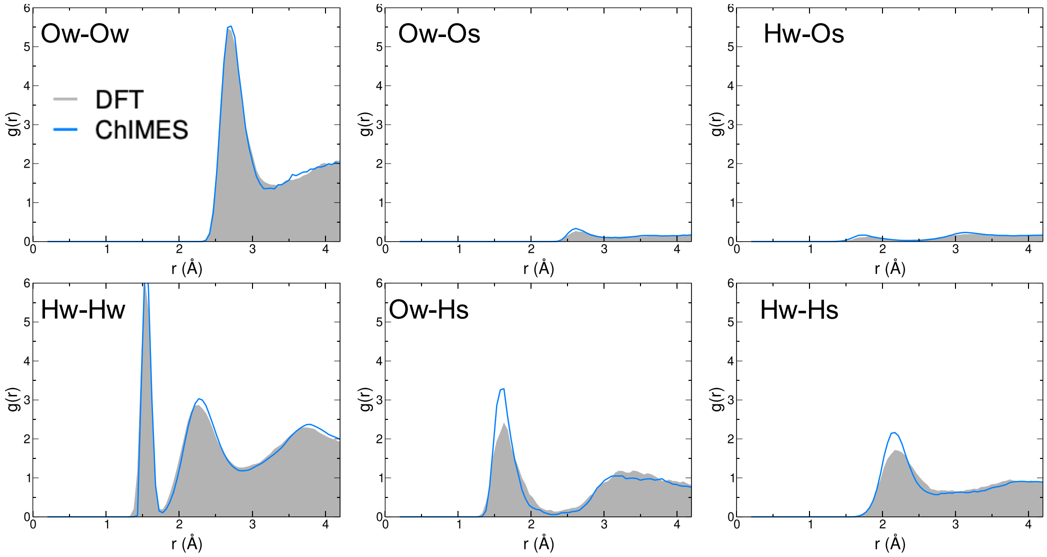

Figure S4: Predicted radial distribution functions, g(r), obtained from DFT and ChIMES for O–O, H–H, and H–O pairs in a system with 75% OH coverage and L = 20Å. The subscripts "w" and "s" indicate atoms belonging to the "water" and "surface", respectively.

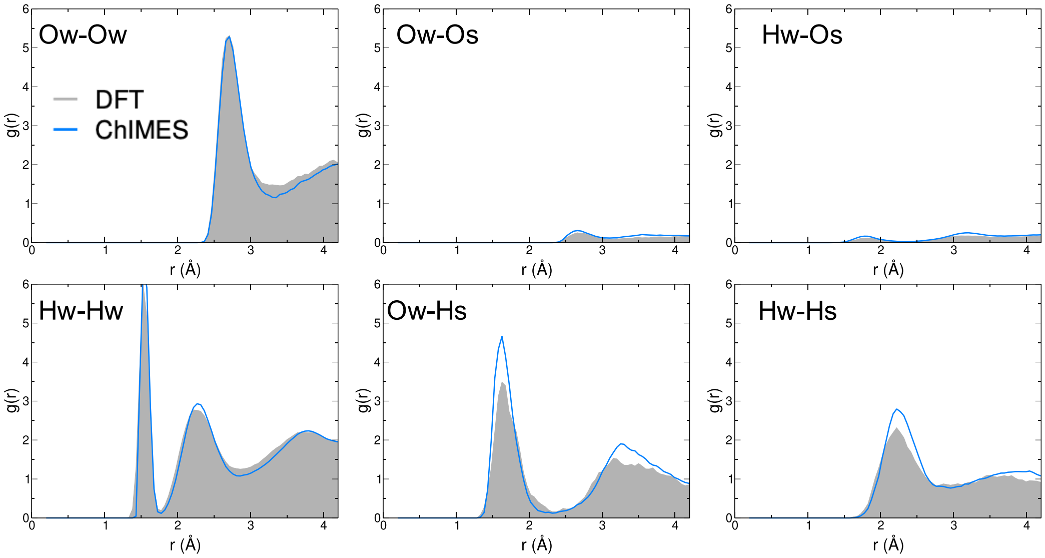

Figure S5: Predicted radial distribution functions, g(r), obtained from DFT and ChIMES for O–O, H–H, and H–O pairs in a system with 50% OH coverage and L = 20Å. The subscripts "w" and "s" indicate atoms belonging to the "water" and "surface", respectively.

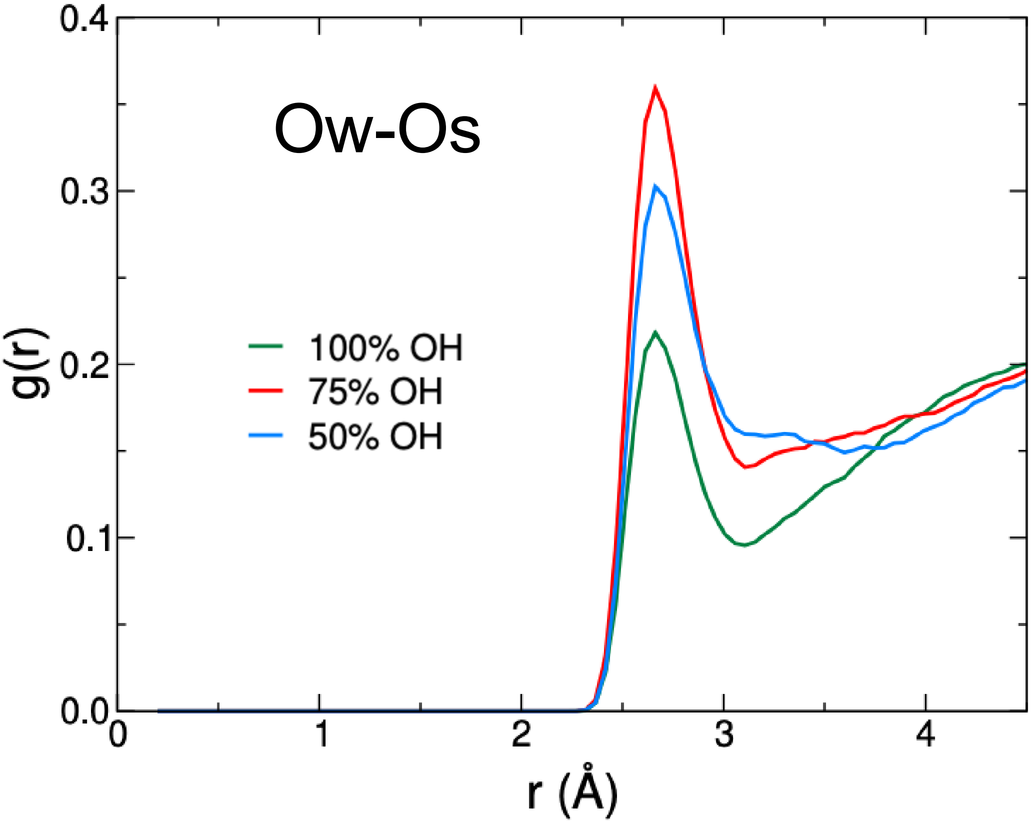

Figure S6: Predicted radial distribution functions, g(r), obtained from ChIMES for Ow–Os in a system with L = 20Å and varying OH coverages. The subscripts "w" and "s" indicate atoms belonging to the "water" and "surface", respectively.
